# Modeling potential risk areas of *Orthohantavirus* transmission in Northwestern Argentina using an ecological niche approach

**DOI:** 10.1186/s12889-023-16071-2

**Published:** 2023-06-26

**Authors:** Walter R. López, Mariano Altamiranda-Saavedra, Sebastián D. Kehl, Ignacio Ferro, Carla Bellomo, Valeria P. Martínez, Mario I. Simoy, José F. Gil

**Affiliations:** 1 Instituto de Investigaciones de Enfermedades Tropicales (IIET), Universidad Nacional de Salta (UNSa), Sede Regional Orán, A4400 Salta, Argentina; 2 Grupo de Investigación Bioforense, Facultad de Derecho Y Ciencias Forenses, Tecnológico de Antioquia Institución Universitaria, Antioquia, Colombia; 3 Instituto Nacional de Enfermedades Infecciosas (INEI), Administración Nacional de Laboratorios E Institutos de Salud (ANLIS) “Dr. C. G. Malbrán”, Buenos Aires, Argentina; 4 Instituto de Ecorregiones Andinas (INECOA), Consejo Nacional de Investigaciones Científicas Y Técnicas (CONICET), Universidad Nacional de Jujuy (UNJu), San Salvador de Jujuy, Argentina; 5 Instituto de Investigaciones en Energía No Convencional (INENCO), Consejo Nacional de Investigaciones Científicas Y Técnicas (CONICET), Universidad Nacional de Salta (UNSa), A4400 Salta, Argentina; 6 Instituto Multidisciplinario Sobre Ecosistemas Y Desarrollo Sustentable (UNCPBA - CICPBA), Tandil, Argentina; 7 Cátedra de Química Biológica Y Biología Molecular de La Facultad de Ciencias Naturales, Universidad Nacional de Salta, A4400 Salta, Argentina

**Keywords:** *Orthohantavirus*, Ecological Niche Modeling, Risk map, Reservoirs, *Oligoryzomys*, *Calomys*, Northwestern Argentina

## Abstract

**Background:**

Hantavirus Pulmonary Syndrome (HPS) is a rodent-borne zoonosis in the Americas, with up to 50% mortality rates. In Argentina, the Northwestern endemic area presents half of the annually notified HPS cases in the country, transmitted by at least three rodent species recognized as reservoirs of *Orthohantavirus*. The potential distribution of reservoir species based on ecological niche models (ENM) can be a useful tool to establish risk areas for zoonotic diseases. Our main aim was to generate an *Orthohantavirus* risk transmission map based on ENM of the reservoir species in northwest Argentina (NWA), to compare this map with the distribution of HPS cases; and to explore the possible effect of climatic and environmental variables on the spatial variation of the infection risk.

**Methods:**

Using the reservoir geographic occurrence data, climatic/environmental variables, and the maximum entropy method, we created models of potential geographic distribution for each reservoir in NWA. We explored the overlap of the HPS cases with the reservoir-based risk map and a deforestation map. Then, we calculated the human population at risk using a census radius layer and a comparison of the environmental variables’ latitudinal variation with the distribution of HPS risk.

**Results:**

We obtained a single best model for each reservoir. The temperature, rainfall, and vegetation cover contributed the most to the models. In total, 945 HPS cases were recorded, of which 97,85% were in the highest risk areas. We estimated that 18% of the NWA population was at risk and 78% of the cases occurred less than 10 km from deforestation. The highest niche overlap was between *Calomys fecundus* and *Oligoryzomys chacoensis*.

**Conclusions:**

This study identifies potential risk areas for HPS transmission based on climatic and environmental factors that determine the distribution of the reservoirs and *Orthohantavirus* transmission in NWA. This can be used by public health authorities as a tool to generate preventive and control measures for HPS in NWA.

**Supplementary Information:**

The online version contains supplementary material available at 10.1186/s12889-023-16071-2.

## Background

Hantavirus pulmonary syndrome (HPS) is a zoonotic disease endemic to the Americas caused by viruses of the *Hantaviridae* family, *Mammantaviridae* subfamily, *Orthohantavirus* genus, widely known as Hantaviruses [1]. These viruses are transmitted to humans through the inhalation of viral particles contained in excretions of different infected rodent species belonging to the Cricetidae family and the Sigmodontinae and Neotominae subfamilies, which act as natural reservoirs. Additionally, although not frequent, person-to-person transmission as well as through rodent bites has been reported [2, 3]. The symptomatic manifestations of HPS can be non-specific febrile syndrome, in its mildest form, or progress to the most severe manifestation with acute respiratory failure and cardiogenic shock [4]. This disease is a public health concern because of the high mortality rate (between 30 to 50%), the lack of specific treatment, and the difficult early diagnosis due to the similarity with other diseases (arboviruses, spotted fever, COVID-19, leptospirosis, hemorrhagic fever arenavirus and others non-specific infections) [3].

**Fig. 1:**
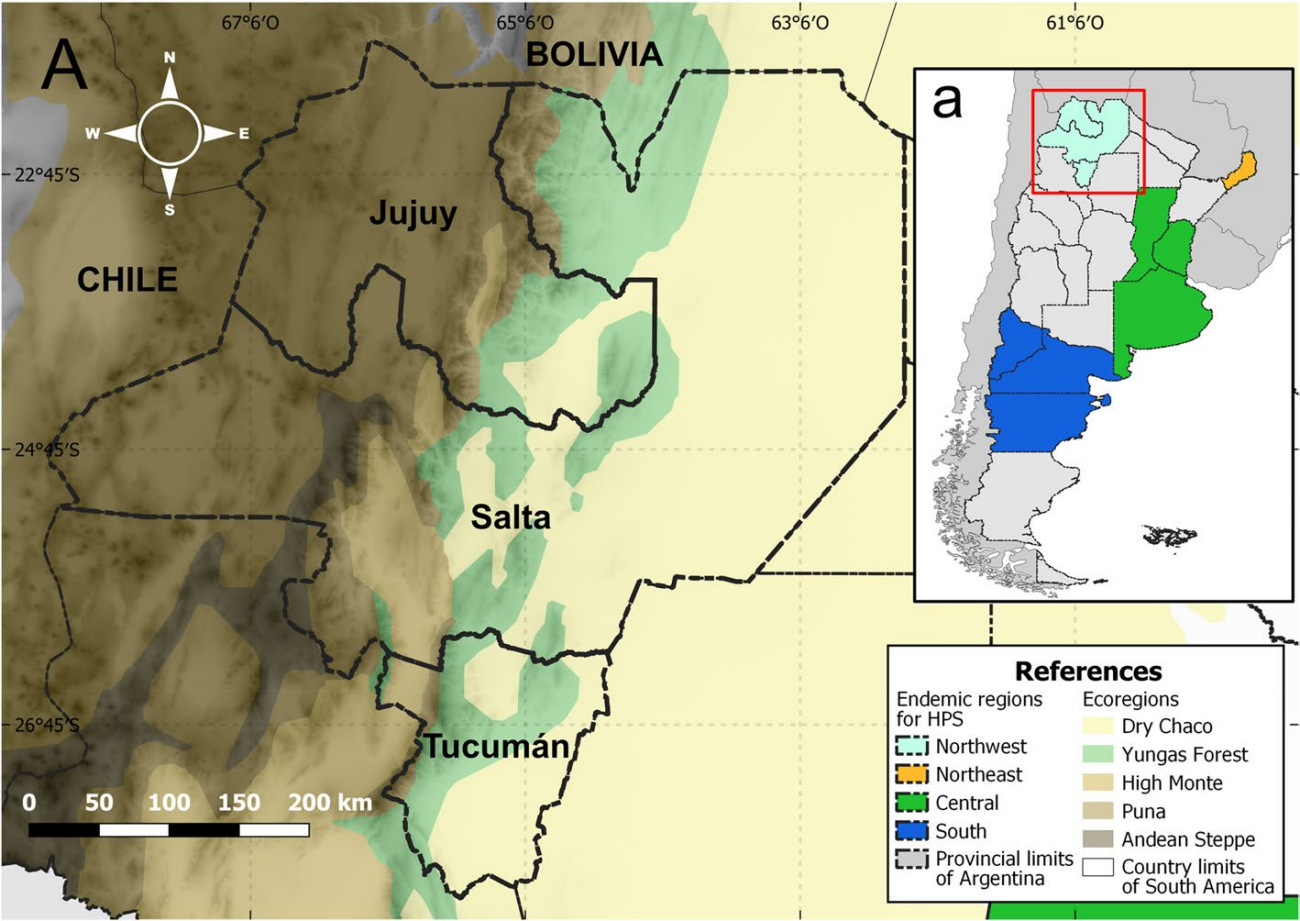
Study area. The Northwestern Argentina Hantavirus Pulmonary Syndrome (HPS) endemic area, which induces the eastern half of Jujuy, Salta, and Tucumán provinces (A). The input box shows the four endemic areas for the HPS in Argentina (a). This figure was created in QGIS V.3.20.2, using free and freely available shapefiles and images (Additional file 1)

According to the Pan American Health Organization, the countries with the highest number of HPS cases in South America are Brazil and Argentina [5]. To date, at least 2 species of pathogenic *Orthohantavirus* that have been recognized by the International Committee of Viral Taxonomy were reported in Argentina: *Andes orthohantavirus*, with several viruses in the 4 endemic regions of the country (Fig. 1): Orán virus (ORNV), Bermejo virus (BERV) and Buenos Aires virus (BAV) in Northwest Argentina (NWA: including Salta, Jujuy and Tucumán provinces), Juquitiba virus and Lechiguanas virus (LECV) in Northeast Argentina, BAV, LECV and Plata virus in Central Argentina and Andes virus (ANDV) in South Argentina; and the second species *Laguna Negra orthohantavirus* in the Northwest endemic region, in which only Laguna Negra virus (LNV) occurs [6, 7]. The highest incidence of cases in the country is associated with *Andes orthohantavirus*, while *Laguna Negra orthohantavirus* was associated with only a few cases. Historically, the Northwestern and Central regions have reported the highest number of HPS cases, 49%, and 36% respectively, of all reported cases in the country followed by the Southern region (14%) and finally the Northeast region with only a few cases [8].

Despite being the area with the highest HPS incidence in Argentina, several aspects related to *Orthohantavirus* transmission in the NWA region are still poorly investigated. Previous studies in the region have determined that *Oligoryzomys chacoensis* and *O. flavescens occidentalis* (west clade) [9], mainly captured in rural peridomestic habitats, are involved in the transmission of ORNV and BERV to humans [10–12]. Also, *Calomys fecundus* was identified as a reservoir of LNV in Yuto, Jujuy province [7]. However, in NWA there is still a nomenclature uncertainty for *C. fecundus*, which is currently considered a junior synonym of *C. boliviae* by some authors [13]. Although there is recent evidence suggesting that they are distinct species, the lack of genetic studies for *C. boliviae* does not allow us to make advances on this issue [13, 14].

To estimate the risk of infection for different human populations, it is necessary to understand which environmental factors shape the geographic distribution of reservoirs involved in *Orthohantavirus* transmission. The ecological niche models approach (ENM) provides a useful tool to identify the relationship between environmental or climatic factors and the distribution of organisms [15]. Therefore, it is possible to model the relationships between organisms associated with zoonotic diseases and the environmental setting where they live to make predictive maps of occurrences and risk of disease. Indeed, the ENM tool has been applied to estimate areas of environmental suitability for reservoirs and vectors, the effect of climate change on their distribution, and the risk of disease transmission [16]. Particularly, this approach has been useful to understand the relationships between Hantavirus and its reservoirs [17, 18]. In Argentina, the ENM approach was applied to determine the potential distribution of *O. longicaudatus* to explore the difference in distribution between subspecies of *O. flavescens*, and to determine risk areas of *Orthohantavirus* infection in southern Argentina [9, 19, 20].

In this context, this work aimed to generate potential distribution models for the three rodent species reservoirs of pathogenic *Orthohantavirus* in the NWA endemic region. We also sought to identify environmental and climatic variables that affect these potential distributions and estimate the degree of niche overlap between these three species. In addition, from these models, we sought to build a risk map of HPS occurrence, estimate the population at risk and analyze the concordance of the risk map with the historical cases of HPS in NWA. Also, we analyzed the possible spatial correlation between the distribution of HPS cases and the presence of nearby deforestation. Finally, the possible effect of the latitudinal variation of bioclimatic variables on the presence and frequency of cases was explored.

## Methods

### Study area

The determined study area was Northwestern Argentina in South America, an endemic region for HPS comprising the lowlands and foothills of Salta, Jujuy, and Tucumán provinces between latitudes 21.8°- 28°S and longitudes 62.32°- 68.20°O (Fig. 1). There are five ecoregions present in these provinces. The western half of the region is dominated by a desert mountain landscape with three ecoregions: The Puna, a Highland Plateau, the High Andean Steppe on the mountain tops, and the Monte Desert in the valleys. Conversely, the eastern half of the studied area is a subtropical forest with two ecoregions: The Yungas rainforest ecoregion on the eastern slopes of the Andes and the Dry Chaco ecoregion, a savanna-like plain (Fig. 1). The sharp contrast in climatic conditions due to sudden changes in altitude determine abrupt changes in rodent species distribution [21].

### Dataset of rodent species presence

We obtained occurrence data from different sources, for the three rodents known to be Hantavirus reservoirs: *O. chacoensis*, *O. f. occidentalis,* and *C. fecundus*. The presence areas (PA; polygons) for these rodents were built based on a merge of the distribution proposals by a) Mammals of South America [22] b) the International Union for Conservation of Nature (IUCN), and c) “Sociedad Argentina para el Estudio de los Mamíferos” (SAREM) (see Additional file 1). Also, for each species, occurrences 50 km away or more from the PA were considered outliers and removed from the database to avoid including occurrences outside of the species distribution range. Rivera et al., [9] found cryptic species within the *O. flavescens* complex based on molecular evidence and suggested that the western Argentinean clade corresponds to *O. f. occidentalis*; thus, some authors followed this criterion to consider *O. occidentalis* as a valid species [23]. Therefore, the occurrence data of *O. flavescens* that were within the PA of *O. f. occidentalis* were included in the study.

Besides, Salazar Bravo et al. [24], based on molecular analysis of mitochondrial genes, suggested that the name *C. fecundus* should be applied to the Yungas ecoregion populations of NWA and Bolivia, but then without further evidence, he listed *C. fecundus* as a junior synonym of *C. boliviae* in a recent checklist of South American Mammals [22]. Furthermore, Pinotti et al. [13], based on molecular and potential distribution approaches, suggested that the Bolivian Montane Dry Forest could have acted as a barrier favoring the differentiation between *C. boliviae* and *C. fecundus*. In the same way as for *O. f. occidentalis*, occurrence data for *C. boliviae* that were within the PA of *C. fecundus*, were included in the study.

We searched for rodent occurrence data (*O. chacoensis*, *C. fecundus*, *O. f. occidentalis*, *O. flavescens,* and *C. boliviae*) in the following databases of scientific literature: PubMed (https://pubmed.ncbi.nlm.nih.gov/), Scielo (https://scielo.org/) and Google Scholar (https://scholar.google.es/schhp?hl=es) (access to data in January and February 2021). Also, we used the Global Biodiversity Information Facility (GBIF) database with RGBIF. To improve the database in our studied region we included a revised specimen based taken from the museum collection “Colección Mamíferos Lillo” (CML) at the National University of Tucumán (see Additional file 2).

We then filtered the distribution record data obtained from RGBIF to prevent problematic records using the CoordinateCleaner R package. Additionally, we eliminated all records without geographic coordinates from the database, as well as duplicated ones (i.e., same geographic coordinates), so we kept only one presence record for each species per location. Finally, we obtained records from 1818 to 2016 and constructed a database with geographic coordinates, references, sampling dates, and type of traps used (Additional file 2).

### Climatic data

We obtained high-resolution raster layers of 19 climatic variables derived from spatial interpolation of temperature and precipitation, containing average values for the period 1979–2013, from the CHELSA website [25]. We also used land cover layers downloaded from the National Mapping Organizations (https://globalmaps.github.io/glcnmo.html). This geospatial information classifies the status of the land cover of the whole globe into 20 categories. Additionally, we obtained the Normalized Difference Vegetation Index (NDVI) from the R package MODIStsp, including the years 2000 to 2012. For NDVI, the maximum, minimum, median, and range were calculated by each grid cell considering the entire time period. All variables were resampled at a resolution of 2 km using the nearest neighbor and bilinear methods for land cover and the rest of the variables, respectively using the R package raster.

To eliminate overfitting and unnecessary complex relationships between climatic variables, we carried out two tests using all variables: A Pearson correlation test, to determine which were correlated, and a Jackknife analysis to determine the degree of individual contribution to a general model. From the groups of correlated variables, with values equal to or greater than 0.8 in the Pearson correlation test, we selected only those variables that presented the highest contribution values in the Jackknife analysis and discarded the remaining ones. The mean annual temperature (Bio1) and annual precipitation (Bio12) were included in almost all the sets of variables, and we eliminated those correlated with them because these two variables are considered of utmost biological importance [26–28]. Based on this approach, we constructed 3 sets of environmental variables of varying complexity by each rodent species (Additional file 1: supple Table A1 and Fig. A1).

### Ecological niche model generation

We used the R package KUENM V.1.1.5 for the generation of the ENM. This package uses the MaxEnt modeling algorithm and largely automates the calibration, evaluation, and transfer steps of the ENM [29, 30]. For the model calibration, we defined an accessible area (M) for each rodent species. The M area represents the hypothetical historical accessible area for the species [31]. The knowledge of rodent dispersal capacity is important to define the M area, but there is no available data on this aspect of rodent biology in Northwestern Argentina. Thus, we built the M areas using the ecoregions where each species occurs based on their point presence location [32]. Then, we organized occurrence data into three data sets by species: 1) All occurrence location records (100%), 2) a training dataset that contained 80% of the randomly selected occurrence records, and 3) a test data set that contained the remaining 20% of the location records.

For each species, we created 1581 candidate models resulting from the combination of three parameters: 1) 3 sets of environmental variables, 2) 17 values of the regularization multiplier (0.1–1 at intervals of 0.1, 2–6 at intervals of 1, 8, and 10) and, 3) all the 31 possible combinations of the 5 features classes (linear = l, quadratic = q, product = p, threshold = t and hinge = h). These parameters were the same for each rodent species in order to make the models comparable. We evaluated the performance of each candidate model according to the following three criteria: a) the statistical significance of the partial Receiver Operating Characteristic curve (partial ROC, with 500 interactions and 50 percent of data for bootstrapping), b) omission rates below 5% and c) the Akaike Information Criterion corrected for small sample size (AICc) considering the difference between the best model with the remaining candidate models (delta AICc) [33]. First, we selected the models according to partial ROC; then, we reduced the number of models to include only those that met the omission rates, and finally, among these sets of remaining models, we selected those with less than 2 delta AIC values as final models [30].

For the selection of each final model, we produced 10 replicates with logistic and raw outputs to facilitate their interpretation and to avoid the effect of the other covariates in the visualization. We used the median of these outputs for visualization and further analysis. Then, we transferred these models outside area M to all of South America. To choose the type of model output (extrapolation, clamping, or no extrapolation), we assigned to each response variable the type of behavior to which it best adjusted and selected the behavior of the models based on the sum of the importance (percentage of contribution) of the response variables [34].

We developed a Movility-Oriented Parity (MOP) analysis to assess the extrapolation map in the prediction of the suitable area by each rodent species. This analysis quantifies the environmental similarity between the calibration and projection areas by highlighting areas where strict extrapolation occurs [34]. To exclude areas with the lowest analog climates, we used the MOP outputs of 10% or higher as a mask layer and cropped the raw projection outputs selected for each rodent species. Then, using QGIS V.3.20.2, we reclassified the raw maps generated into binary maps by a threshold that omits all regions with habitat suitability below the values for the lowest 10% of occurrence records. These last maps represent the potential distribution of each reservoir in the study area.

### Niche overlapping

We made a niche overlap analysis for the three rodent reservoirs, using Niche Analyst 3.0 [35]. An environmental space was created from the first three main principal components that contained 90% of the variation in the 19 CHELSA climatic variables, the NDVI (maximum, minimum, mean, and range), and a land cover layer, in a polygon that contained the M areas of the rodent species. We generated simulations of occupied virtual niches from the total occurrence points for each rodent species and calculated the minimum-volume-ellipsoid. From each occupied niche, we obtained its attributes and quantified the overlap volume (niche similarity) for the three interacting species, through the Jaccard index (0 to 1).

### HPS cases frequency and incidence

We obtained the HPS cases frequency by province, department, and fine geographic scale (locality, town, and farm) of the Northwestern region from the “Instituto Nacional de Enfermedades Infecciosas Malbrán—Administración Nacional de Laboratorios e Institutos de Salud Dr. Carlos Malbrán” (INEI ANLIS). We recorded all cases since the first reported case in NWA in 1997 to 2019. We considered a confirmed HPS case to be any patient who attended the health system and was diagnosed by serological and/or molecular detection techniques for *Orthohantavirus*. Since the population data by town or farm was not available, we had to group the mean yearly incidence at the department level (the third political level division). The population for each department was estimated by means of a linear extrapolation of the censuses of the years 1991, 2001, and 2010 from “Instituto Nacional de Estadística y Censos de la República Argentina” (INDEC) (census of the years 1991, 2001 and 2010; see Additional file 1).

### Risk map, deforestation, and population at risk

To build disease transmission risk maps, we used the raw outputs of the best models cropped with the MOP data to compute an average of the three models. Then, we used the k-means unsupervised classification algorithm to classify the data into four categories of environmental suitability. In this way, the resulting map shows four categories according to the level of risk, defined based on the overlapping areas of suitability for rodent reservoir species: low risk, medium risk, high risk, and very high risk.

We estimated the population at risk using: a) the very-high and high strata of the risk map described in the previous paragraph, b) the population of the census radius of the National Institute of Statistics and Censuses (INDEC; census of the year 2010) and c) the layer of departments (third political level division) of the study area with a mean yearly incidence greater than 1/100,000 inhabitants. The departments of Yerba Buena and Burruyacu had 1 and 2 cases respectively, both in the province of Tucumán. These departments were included, given that epidemiological studies confirmed that they were autochthonous cases [7, 36].

In addition, we extracted the mean values of the risk map for each department using the QGIS zonal statistics plugin. This means that the level of risk was compared with the mean yearly incidence of HPS using Spearman’s correlation. Considering the area with a very high risk on the map, we analyzed the behavior of the bioclimatic variables according to the latitude variation and compared it with the values of the bioclimatic variables in sites with confirmed HPS cases occurrence. Additionally, we explored the question of whether the behavior of the variables fits a polynomial model.

On the other hand, we obtained a layer of deforestation areas of the study area from the Spatial Data Infrastructure of the province of Salta (http://geoportal.idesa.gob.ar/), to explore whether the sites with the presence of HPS cases are close to deforestation. In the rainforest ecoregion, most of the deforested areas are used for agricultural purposes, predominantly crops of sugar cane, bananas, and citrus [37]. Also, in the dry Chaco region, a complex process initially associated with livestock was identified. This leads from dry forests to pastures, then to monocultures, and later to monoculture and double cropping systems, where processes of expansion, substitution, and intensification occur [38]. For the analysis, we generated buffers of 5 km and 10 km from the layer of HPS cases. We only counted the sites that overlapped with the clearings and occurred between 1997 and 2019.

## Results

We obtained 106 occurrence data for *O. chacoensis*, 99 for *O. f. occidentalis,* and 174 for *C. fecundus*. The distribution of *O. chacoensis* included northern Argentina, southern Bolivia, and central Paraguay. The distribution of *O. f. occidentalis* covers northern and central Argentina, as well as central and southern Bolivia. Finally, the distribution of *C. fecundus* was the most restricted one, in northern Argentina and southern Bolivia occupying the forest strip on the eastern slopes and lowlands close to the Andes (Additional file 1: supple Fig. A2, A3 and A4).

The four variables that contributed most to the models were: For *O. chacoensis*: temperature seasonality (Bio4), precipitation seasonality (Bio15), isothermality (Bio3), and mean monthly precipitation amount of the warmest quarter (Bio18); for *O. f. occidentalis*: precipitation seasonality (Bio15), mean annual temperature (Bio1), NDVI mean and annual range of temperature (Bio7); and for *C. fecundus*: precipitation seasonality (Bio15), the annual range of temperature (Bio7), mean annual temperature (Bio1) and annual precipitation amount (Bio12) (Additional file 1: supple Table A2). In the transfer, the response curves of the selected models suggested that their behavior outside the M area better fits the “extrapolation” type of transfer for most of the variables (Additional file 1: supple Fig. A5-A7).

The model’s evaluation allowed us to select only one best final model for each of the three reservoirs (Additional file 1: supple Table A3 and A4). For the three reservoirs, MOP analysis of the spatial projection showed that the environments represented were similar to the calibration areas to a large extent. Strict extrapolation areas for *O. chacoensis* were presented in southern Venezuela, eastern Colombia, western and eastern Ecuador, western, central, and southeastern Peru, central Bolivia, and northern Chile. For *O. f. occidentalis*, the strict extrapolation areas were in central Venezuela, western and central Ecuador, a large part of central and southern Peru, and northern and southern Chile. Finally, *C. fecundus* displayed strict extrapolation areas in central and southern Guyana and Venezuela, isolated areas of Colombia, western and eastern Ecuador, western and southeastern Peru, central Bolivia, northern and southern Chile, and northern Brazil (Additional file 1: supple Fig. A8). It should be noted that the MOP analysis does not suggest, for any of the species, eliminating sections of the transfer areas outside the M area, within our study area (Additional file 1: supple Fig. A8).

**Fig. 2:**
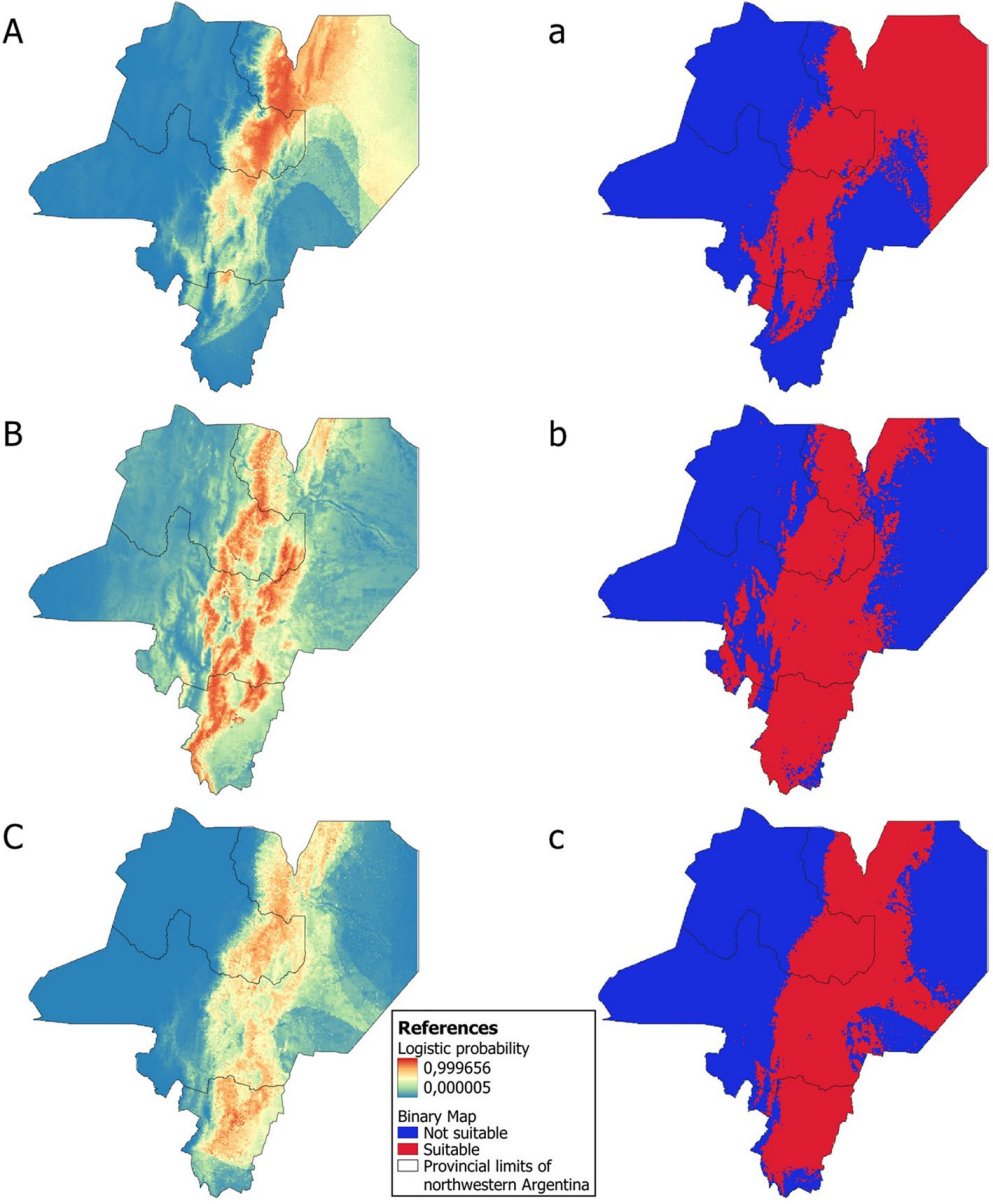
Logistic outputs and binary maps for the three reservoirs. *O. chacoensis* (**A, a**), *O. f. occidentalis* (**B, b**) and *C. fecundus* (**C, c**). This figure was created in QGIS V.3.20.2, using free and freely available shapefiles (Additional file 1)

In general, the best models for the three reservoirs showed areas of high environmental suitability in the north and center of Salta and Tucumán, and in the southeast of Jujuy. The binary maps indicated that the potential distribution of *O. chacoensis* covers most of the Dry Chaco ecoregion, east and northeast of Salta, and some regions of the Yungas rainforest ecoregion extending southwards as far as northern Tucumán province; while *O. f. occidentalis* mainly covers the Yungas rainforest encompassing a large part of central Salta province and almost all of Tucumán province. Finally, *C. fecundus* potential distribution covers a large area in both ecoregions (Fig. 2).

**Fig. 3:**
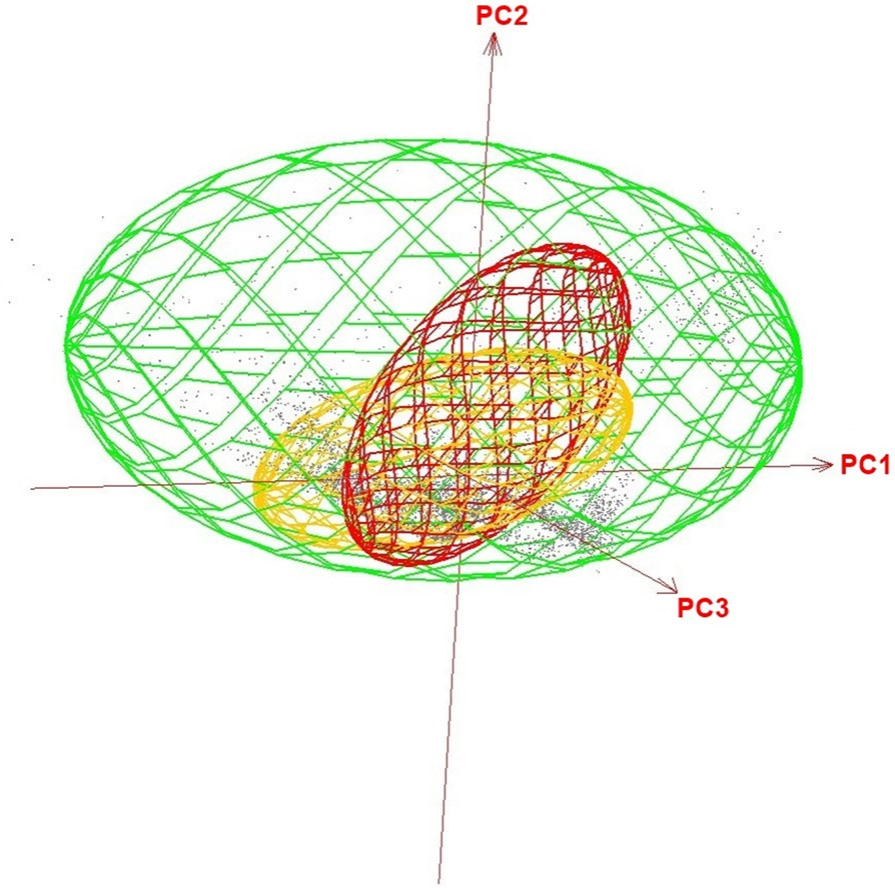
Minimum volume ellipsoids, in environmental three-dimensions, for the three reservoirs. *O. chacoensis* (orange ellipsoid), *O. f. occidentalis* (green ellipsoid), and *C. fecundus* (red ellipsoid). Gray points represent the environmental space and PC1, PC2, and PC3 represent the three principal components of the 24 environmental variables

The Jaccard index yielded low values of niche overlap of the three rodent species analyzed. The highest niche overlap was between *C. fecundus* and *O. chacoensis* with a value of 0.26, while between *O. chacoensis* and *O. f. occidentalis* it was 0.15 and the lowest value found was between *C. fecundus* and *O. f. occidentalis* with a value of 0.11. The highest niche amplitude was reached by *O. f. occidentalis,* compared to *O. chacoensis* and *C. fecundus* (Fig. 3).

**Fig. 4:**
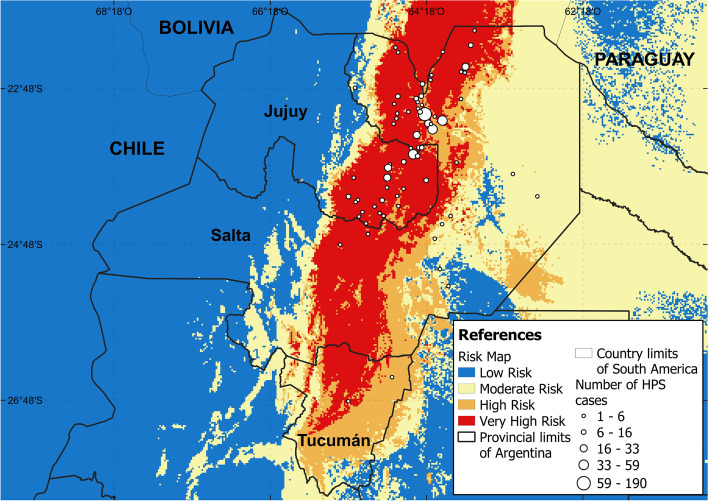
Risk areas of *Orthohantavirus* transmission in the study area. This figure was created in QGIS V.3.20.2, using free and freely available shapefiles (Additional file 1)

The risk map indicated high and very-high risk of transmission of O*rthohantavirus* in the Yungas rainforest and part of the Dry Chaco ecoregions, which correspond to the north and center of Salta and Tucumán provinces as well as southeast of Jujuy province (Fig. 4), covering a great part of the study area. A large part of the Dry Chaco ecoregion was classified as moderate risk, which includes mostly the eastern part of Salta province. Considering the human population, it was estimated that a total of 600,791 people were at risk (18% of the total population of the 3 provinces).

A total of 945 HPS cases occurred during the study period, of which 904 had geographic information up to at least the department level. Considering the area that includes the departments with case reports, the global mean yearly incidence of HPS was 2.31/100,000 inhabitants. The HPS mean yearly incidence by department varied between 0.01 and 14.32/100,000 inhabitants and the departments with a mean yearly incidence greater than 1/10,000 inhabitants were: Orán, San Martín, Anta, Iruya, Rivadavia, and Santa Victoria in Salta province; Libertador General San Martín, Santa Bárbara, San Pedro and San Antonio in Jujuy province. Ten of the recorded cases corresponded to travelers of Bolivian origin.

**Fig. 5:**
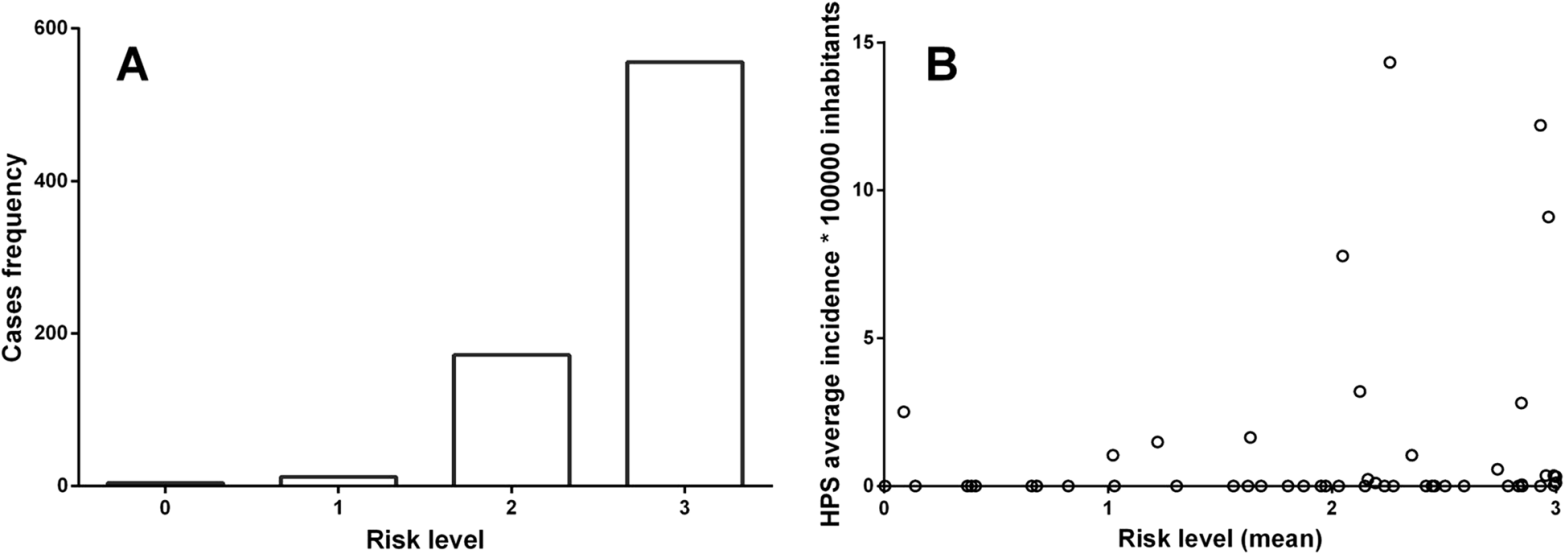
Case frequency and mean yearly incidence of HPS versus risk level. **A** shows the frequency of cases as a function of the risk level derived from the model and **B**) HPS mean yearly incidence by department as a function of the average risk level obtained from the model in raster format (average of the pixels included in each department)

Besides, a total of 744 cases could be identified at a finer scale (locality, town, or farm level). These cases were distributed in 69 sites, of which 40 belong to the Yungas rainforest (512 cases; 68.81%), 28 to the dry Chaco (231 cases; 31.04%), and 1 to the Puna (1 case; 0.13%) ecoregions. Considering the risk strata obtained by our map, we were able to observe that 47 (74.73% of cases), 15 (23.12% of cases), 4 (1.61% of cases), and 3 (0.54% of cases) of the sites corresponded to strata 3, 2, 1 and 0 respectively (Fig. 5A). Furthermore, when exploring the correlation between the HPS mean yearly incidence per 100,000 inhabitants by department with respect to the average of the risk strata (average by department of the pixels in the raster format risk map), a statistically significant positive correlation emerged (*r* = 0.43; *p* < 0.001). In addition, all the departments with mean yearly incidence values greater than 1/100,000 inhabitants are found in areas whose strata average is greater than one (Fig. 5B). Regarding deforestation, 52 sites (668 cases) and 69 sites (744 cases) were found within a radius of 5 km and 10 km from some deforestation, respectively.

The variation of Bio12 against latitude showed an oscillation of the precipitation values with higher values in the north and south of the study area. The sites with the highest number of HPS cases (localities, towns, and farms) are concentrated in the northern area. In the south, the precipitation conditions in Burruyacú and Yerba Buena departments, where HPS cases were recently detected, are similar to the amount of precipitation that occurs in the north, where most cases are concentrated. Likewise, when analyzing the Bio15 variable, the lower seasonal variations in rainfall were associated with the occurrence of HPS cases. It should be noted that both the variables Bio12 (R^2^ = 0.29) and Bio15 (R^2^ = 0.54) were adjusted to third-order (cubic) polynomial models.

## Discussion

Almost 30 years after the identification of HPS in Northwestern Argentina, this is the first risk map built to warn health authorities about high-risk regions for *Orthohantavirus* infections. This risk map predicts very well the vast majority of cases that have already occurred, despite having been generated from the information on the presence of the reservoirs, and shows us a region with apparent environmental suitability for viral transmission that should be at least less epidemiologically monitored.

As for many organisms, the rodents’ abundance can be affected by temperature and precipitation, since this can favor the growth of vegetation and access to food and shelter, thus favoring their reproduction and survival [26–28]. Also, these ecologically relevant climatic factors frequently impose limits to species’ physiological tolerance and thus their geographical distribution. However, the effect of the climatic variables on all rodent species is not the same [39]. For the three rodents species, the temperature-associated variables with the greatest contribution to the models were temperature seasonality, isothermality (*O. chacoensis*), and mean annual temperature (*O. f. occidentalis* and *C. fecundus*). We have calculated that the average annual temperatures for places with the presence of these three rodents vary between 14.7 °C to 25.7 °C, 6.85 °C to 23.2 °C and -1.4 °C to 27,7 °C respectively [25]. These value ranges in NWA are observed principally in subtropical areas that include the Yungas rainforest and Dry Chaco ecoregions, markedly different from that expected for *O. longicaudatus*, which is also a reservoir of *Orthohantavirus* in southern Argentina [19].

Regarding the rainfall, our models for the three rodent species showed that rainfall seasonality had a greater contribution to them. This is probably due to the fact that an increase in the amount of annual precipitation generates greater suitability for rodent species. If this occurs for *Orthohantavirus* reservoirs, the risk of transmission may be increased. Abrupt increases in population density have been reported in Argentina for the *Akodon*, *Calomys*, *Mus,* and *Oligoryzomys* genera, known as “ratadas”, associated with increases in mean annual rainfall [40]. Similar results were previously reported for reservoirs in the southern endemic region [19, 20] and for *C. fecundus* in the NWA endemic region [13].

The contribution of the mean NDVI to the distribution models of *O. f. occidentalis* and *C. fecundus* may be related to the productivity of the ecosystem in the M area. In fact, a previous study carried out in Mexico, in which rodent species were monitored on an altitudinal gradient, highlighted the importance of ecosystem productivity (estimated by NDVI) on the diversity and abundance of rodents [41]. However, when the vegetation cover is preserved and the community reaches its climax state, the diversity of species is greater. Therefore, in an advanced stage of ecological succession, the presence of competitors and predators may maintain reservoir populations at relatively low abundances, which may decrease the dynamics of virus transmission risk [42].

The steep elevation gradient of the Andes mountains appeared as a natural barrier limiting the western distribution of the three species [13, 43]. However, in the eastern half of the study region, we found differences between the potential geographic distributions predicted by the models for the three reservoirs. Although the three species have high environmental suitability in the Yungas ecoregion, *O. chacoensis* shows greater environmental suitability than the others followed by *C. fecundus*, then *O. f. occidentalis* in the dry Chaco [13]. In addition, the niche overlap was quite low between the species, being *O. f. occidentalis* the rodent with the largest minimum volume ellipsoid (Fig. 3), which indicates that this species has great adaptive plasticity [22, 44].

The differences found in the suitable areas between the three reservoirs could have important implications for the occurrence of HPS in the Northwestern Argentina endemic region. According to our results, the two species involved in the transmission of pathogenic *Orthohantavirus*, *O. chacoensis,* and *O. f. occidentalis*, for ORNV and BERV respectively, would have a similar distribution in the Yungas rainforest, but in the Dry Chaco, *O. chacoensis* would have a predominant role. Probably the combined abundance of the two rodent species can generate a greater number of HPS cases, compared to sites where only one of the species is present. On the other hand, *C. fecundus*, carrying LNV has suitable habitats in both ecoregions, but only a few infected rodents and human cases of HPS associated with LNV have been recorded in NWA [7].

Our risk map suggests that the areas with the highest risk of *Orthohantavirus* transmission were mainly the Yungas rainforest and the occidental zone of Dry Chaco. Particularly, our results highlight the north of Salta province and the east of Jujuy province, where indeed the HPS cases are highly clustered [6, 8]. This suggests that our risk map has a very good sensibility in detecting very high-risk areas for *Orthohantavirus* transmission based solely on the reservoir distribution modeling. It is important to highlight that most localities with HPS cases are geographically included in the highest stratum (very high-risk) of the risk map and that mean yearly incidence by department are positively correlated with risk levels (Fig. 5A y B) [8, 45].

**Fig. 6:**
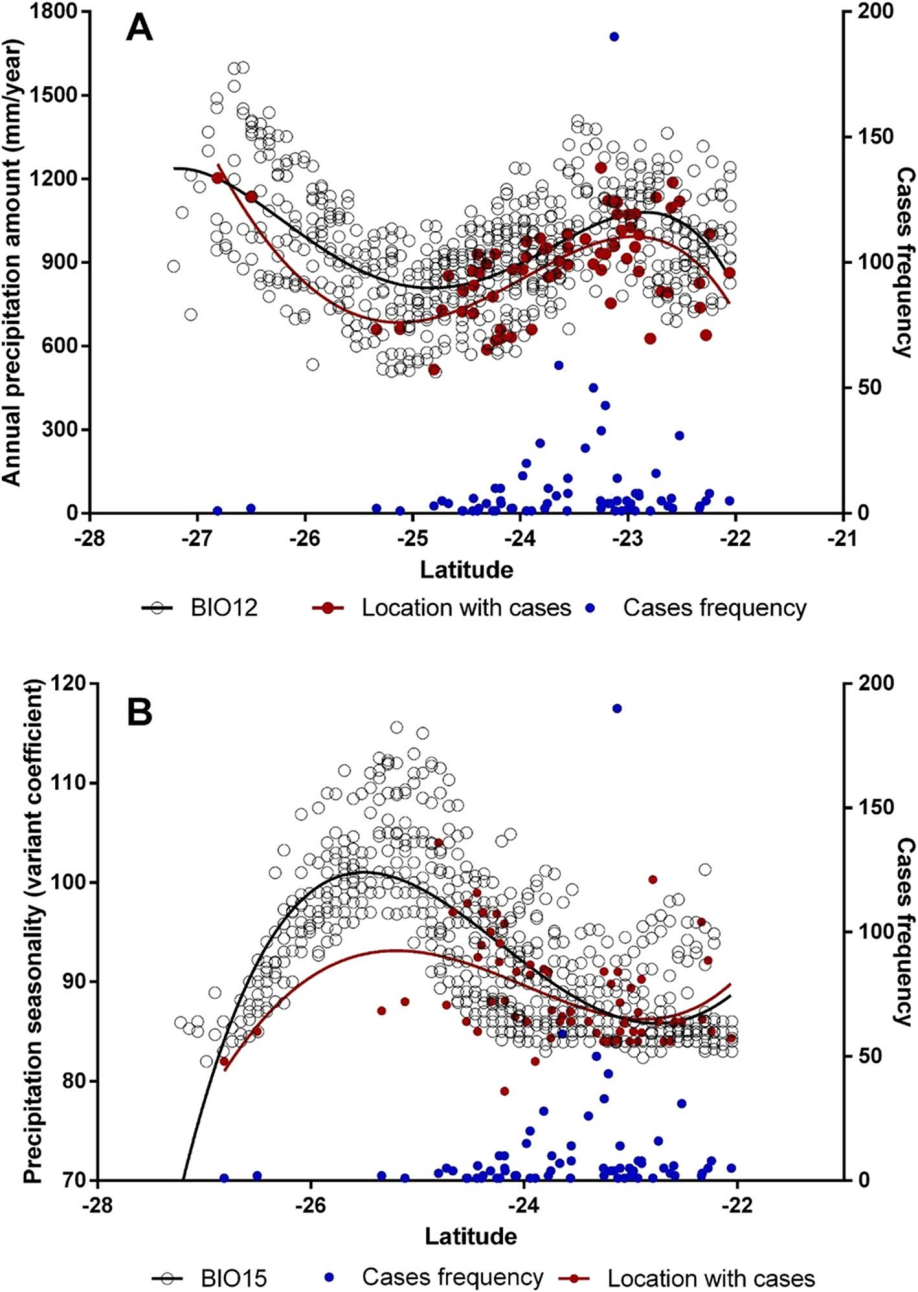
Analysis of the variation of precipitation and latitude. **A** Annual precipitation amount (mm/year) variation versus latitude variation (Left: South; Right: North). The red dots represent the precipitation values of the sites with reported presences according to latitude. Open dots represent the precipitation values of a grid point arranged latitudinal. The blue dots represent the frequency of HPS cases according to latitude (frequency values are shown on the y-axis to the right) and **B**) Precipitation seasonality versus latitude variation. The red dots represent the precipitation seasonality values of the sites with cases presence according to latitude. Open dots represent the precipitation seasonality values of a grid point arranged latitudinally. The blue dots represent the frequency of HPS cases according to latitude (frequency values are shown on the y-axis to the right)

The risk map also predicts areas with high risk where no cases of HPS were reported. However, HPS cases have recently been reported in the southern area of the NWA within the province of Tucumán [7, 36]. This shows that the risk zones predicted by the risk map have the conditions for the maintenance of the *Orthohantavirus* zoonotic transmission cycle and that the illness is probably under-reported. These places had not previously reported cases and are located in the very high-risk and high-risk strata according to the risk map, a zone that has precipitation conditions similar to the northern sector, where most of the cases are concentrated (considering the latitudinal variation; Fig. 6A and B).

We found that localities with the highest number of cases are located in the northern part of NWA and that the HPS cases seem to be associated with high rainfall and low seasonality of rainfall (Fig. 6A and B). In a systematic review, evidence was found for the association of HPS with precipitation and habitat type, and mixed evidence for temperature and humidity [39]. Furthermore, Ferro et al., [45] showed a relationship between HPS cases and lagged rainfall and temperature with a delay of 2 to 6 months in the Argentinean Northwestern region.

Assuming there are no geographic barriers, bioclimatic conditions can affect the presence of the virus in a given place in two main ways: a) indirectly, favoring or limiting the distribution and abundance of reservoirs, and b) directly, by affecting the viability of the virus in the environment. The comparison of the risk areas predicted by our map (constructed from the potential distribution of reservoirs), with the areas where the sustained presence of HPS cases occur, might indicate which are the limiting bioclimatic variables (and the thresholds) for *Orthohantavirus* distribution.

The high-risk areas of *Orthohantavirus* transmission in NWA have a subtropical climate with high temperatures and summer rainfall, which favors the development of vegetation that serves as food and shelter for rodents. This in turn gives rise to population growth of the reservoirs and thus a greater probability of contact between them either in burrows or through fights between adult male rodents, which leads to a higher prevalence of viral infection in these reservoirs. In this context, *Orthohantavirus* transmission to humans commonly occurs in wild or rural places when people either work or carry out recreational activities which normally include trekking, hunting, and fishing. Meanwhile, occupational exposure can include deforestation, agriculture, transport, and military activities [6, 46]. Some HPS cases were also detected in ferrymen (“bagayeros”) that carry out cross-border trade of legal and illegal merchandise. In addition, in rural places, transmission events occur when rodents invade the sheds or warehouses indoors and their excretions are aspirated by people when cleaning the place [6].

Deforestation normally causes structural alteration in the environment that some reservoir rodents can exploit to find shelters/or even migrate to populated rural settlements or urban places. These changes in the environment can include agricultural or livestock exploitation, a combination of the two, or even the disorderly expansion of cities that can put people in contact with the reservoirs. In addition, deforestation and the subsequent change in land use usually bring with it a significant loss of biological diversity [47]. It has been previously reported that the loss of diversity in ecosystems can increase the risk of Hantavirus transmission due to the loss of the dilution effect [48]. This dilution effect consists in ecosystems with high diversity where the reservoir-virus-reservoir contact is hindered by their interaction with other organisms [42, 49, 50]. Particularly, in NWA most sites with HPS cases are at a short distance from deforestation areas. In addition, the largest area with a high risk of transmission of the virus is found in the rainforest region, where after deforestation the cleared areas are mainly destined to agricultural exploitation, which can provide a suitable environment for the maintenance of rodent populations.

In addition, when we talk about the population at risk, we are considering the areas with high risk according to the risk map and the departments with a frequent presence of cases. In our opinion, not the entire area of a department is at risk of transmission. However, due to a matter of proximity, we can think of risk as the possibility that people may be exposed due to the displacement whether for work, recreation, etc.

As limitations of this work, we can mention the difficulty to collect complete information on some cases in the region, including those reported at the beginning of the study period. Additionally, it is important to inquire about unknown hosts and reservoirs in the region, since there is increasing evidence of several animals linked to Hantavirus, such as bats or new rodent species, which may act as hosts and potential reservoirs of these and currently unknown viruses [51]. For example, *C. callosus* and *C. laucha* are reservoirs of LNV in Bolivia and Paraguay respectively. Therefore, our risk map should be updated periodically depending on whether new records and reservoirs for this zoonotic disease are found. We must also consider that the possible low accessibility of people to areas of high environmental suitability for the virus may be generating an overestimation of the population at risk.

## Conclusions

In this work we have advanced in the identification of potential O*rthohantavirus* transmission risk zones. The area of greatest risk has been delimited in a north–south strip that covers the north, center, and south of Salta, the east of Jujuy, and the north and center of Tucumán. Most HPS cases are included in the highest-risk area of our model. The transmission events that occurred in Tucumán province, a place where HPS cases were not normally detected, may be an example that the risk model predicts areas in which transmission can be established, at least temporarily. The temperature, precipitation, and vegetation cover variables could determine the distribution of the reservoirs and the virus in NWA. Based on the model, a significant amount of the population exposed to the risk of transmission is predicted, so it is worth asking whether the number of cases detected by the health system represents only a portion of the population that has been infected with this virus. Moreover, we have found that a large part of the HPS cases have occurred in localities or places close to deforestation zones.

In addition, the niche overlap of the species is low, which leads us to consider the possibility that the differentiation of suitability zones of these rodents may expand the risk areas of virus transmission. Finally, the areas where HPS cases occur are associated with a high amount of precipitation in all seasons. These results are relevant for the local health system, as they can help to plan preventive and control interventions for HPS in northern Argentina. In addition, the potential distribution models of these rodent species that we have generated can also be used to explore areas of *Orthohantavirus* transmission risk in other South America countries bordering Argentina.

## Supplementary Information

**Additional file 1: Supplementary Tables and Figures. Supple Table A1.** Sets of environmental variables used for ecological niche models for three reservoirs. **Supple Table A2.** Values and range of values of maximum environmental suitability of the variables of the best models for the three reservoirs. **Supple Table A3.** Number of models that passed the different evaluation criteria applied in the calibration for the three reservoirs. **Supple Table A4.** Selected models according to the three defined criteria of selection: Partial ROC, Omission Rates, and Akaike Informative Criteria corrected (AICc). **Supple Fig. A1.** Jackknife analysis and Pearson test in M area for the three reservoirs. **Supple Fig. A2.** Points of occurrence of O. chacoensis in South America. **Supple Fig. A3.** Points of occurrence of O. f. occidentalis in South America. **Supple Fig. A4.** Points of occurrence of C. fecundus in South America. **Supple Fig. A5.** Response curve for O. chacoensis. **Supple Fig. A6.** Response curve for O. f. occidentalis. **Supple Fig. A7.** Response curve for C. fecundus. **Supple Fig. A8.** Logistic outputs of best models extrapolated in South America, and MOP analysis for the three reservoirs.

**Additional file 2:** **Excel A1.** Rodents occurrence records.

## Data Availability

All data generated or analyzed during this study are included in this published article [and its supplementary information files].

## Abbreviations

AICc: Akaike Information Criterion Corrected
ANDV: Andes Virus
BAV: Buenos Aires Virus
BERV: Bermejo Virus
CML: Colección Mamíferos Lillo
ENM: Ecological Niche Model
GBIF: Global Biodiversity Information Facility
HPS: Hantavirus Pulmonary Syndrome
INDEC: Instituto Nacional de Estadística y Censos de la República Argentina
INEI ANLIS: Instituto Nacional de Enfermedades Infecciosas Malbrán—Administración Nacional de Laboratorios e Institutos de Salud Dr. Carlos Malbrán
LEC: Lechiguanas Virus
LNV: Laguna Negra Virus
M: Accessible Area
MOP: Mobility-Oriented Parity
NDVI: Normalized Difference Vegetation Index
NWA: Northwestern Argentina
ORNV: Orán Virus
ROC: Receiver Operating Characteristic Curve
PA: Presence areas
Bio1: Mean annual temperature
Bio3: Isothermality
Bio4: Seasonal temperature
Bio7: Annual range of temperature
Bio12: Annual precipitation
Bio15: Precipitation seasonality
Bio18: Precipitation of warmest quarter
