# Supplementary material for: Modeling potential risk areas of Orthohantavirus transmission in Northwestern Argentina using an ecological niche approach
Source: BMC Public Health. 2023 Jun 26;23:1236. doi: 10.1186/s12889-023-16071-2 (PMC10294401; doi:10.1186/s12889-023-16071-2)
Supplement: Supplementary file 1 — Additional file 1: Supplementary Tables and Figures. Supple Table A1. Sets of environmental variables used for ecological niche models for three reservoirs. Supple Table A2. Values and range of values of maximum environmental suitability of the variables of the best models for the three reservoirs. Supple Table A3. Number of models that passed the different evaluation criteria applied in the calibration for the three reservoirs. Supple Table A4. Selected models according to the three defined criteria of selection: Partial ROC, Omission Rates, and Akaike Informative Criteria corrected (AICc). Supple Fig. A1. Jackknife analysis and Pearson test in M area for the three reservoirs. Supple Fig. A2. Points of occurrence of O. chacoensis in South America. Supple Fig. A3. Points of occurrence of O. f. occidentalis in South America. Supple Fig. A4. Points of occurrence of C. fecundus in South America. Supple Fig. A5. Response curve for O. chacoensis. Supple Fig. A6. Response curve for O. f. occidentalis. Supple Fig. A7. Response curve for C. fecundus. Supple Fig. A8. Logistic outputs of best models extrapolated in South America, and MOP analysis for the three reservoirs. [file 12889_2023_16071_MOESM1_ESM.docx]

**Additional file 1**

**Table A1**. Sets of environmental variables used for ecological niche models for the three reservoirs.

| Set of environmental variables | *O. chacoensis* | *O. f. occidentalis* | *C. fecundus* |
| --- | --- | --- | --- |
| 1 | CHELSA: Bio1, 2, 3, 4, 12, 15, 17, 18  Soil Cover | CHELSA: Bio1, 2, 3, 7, 12, 15, 19  Soil Cover | CHELSA: Bio1, 2, 3, 7, 12, 15, 17  Soil Cover |
| 2 | NDVI: mean, rang  Soil Cover | NDVI: mean, rang  Soil Cover | NDVI: mean, rang  Soil Cover |
| 3 | CHELSA: Bio1, 2, 3, 4, 12, 15, 17, 18  NDVI: rang  Soil Cover | CHELSA: Bio1, 2, 3, 7, 12, 15, 19  NDVI: mean, rang  Soil Cover | CHELSA: Bio1, 2, 3, 7, 12, 15, 17  NDVI: mean, rang  Soil Cover |

Note: Bio1: mean annual temperature (x*10, °C); Bio2: mean diurnal range (°C), Bio3: isothermality [(Bio2/Bio7)*100]; Bio4: seasonal temperature (Standard deviation*100); Bio7: annual range of temperature (x*10, °C); Bio12: annual precipitation (mm/year); Bio15: precipitation seasonality (variant coefficient); Bio17: precipitation of driest quarter (mm/quarter); Bio18: precipitation of warmest quarter (mm/quarter); Bio19: mean monthly precipitation amount of the coldest quarter (mm/quarter).

**Table A2**. Values and range of values of maximus environmental suitability of the variables of the best models for the three reservoirs.

| *O. chacoensis* | Bio4 | Bio15 | Bio3 | Bio18 | Bio1 | Soil Cover | Bio17 | Bio12 | Bio2 |  |
| --- | --- | --- | --- | --- | --- | --- | --- | --- | --- | --- |
| Percentage contribution | 22 | 21.9 | 20.7 | 17.7 | 5.7 | 4.9 | 4.2 | 2.1 | 0.7 |  |
| Type of behavior | C | E | E | E | E |  | E | NE | E |  |
| Suitability value or range | 4000 | >120 | 400 - 470 | >600 | >50 | 18, 7 | >100 | <1500 | >60 |  |
| *O. f. occidentalis* | Bio15 | Bio1 | NDVI mean | Bio7 | Soil Cover | Bio12 | Bio3 | NDVI rang | Bio2 | Bio19 |
| Percentage contribution | 35.6 | 21 | 17.8 | 14.9 | 5.3 | 3.1 | 1.2 | 0.8 | 0.4 | 0 |
| Type behavior | E | NE | E | E |  | E | E | E | E | E |
| Suitability range | >60 | -50 - 50 | >2000 | <150 | 18 | >1500 | <400 | - | <80 | - |
| *C. fecundus* | Bio15 | Bio7 | Bio1 | Bio12 | Soil Cover | NDVI mean | Bio17 | Bio3 | NDVI rang | Bio2 |
| Percentage contribution | 33.5 | 20.2 | 13.7 | 13.3 | 5.5 | 4.8 | 3.9 | 3.5 | 0.8 | 0.7 |
| Type behavior | E | E | E | C |  | E | E | E | E | E |
| Suitability range | >120 | 210 | 210 | 700 - 1300 | 18, 9 | >6000 | >80 | <400 | 3000 | >120 |

Note: Bio1: mean annual temperature (x*10, °C); Bio2: mean diurnal range (°C), Bio3: isothermality [(Bio2/Bio7)*100]; Bio4: seasonal temperature (Standard deviation*100); Bio7: annual range of precipitation (x*10, °C); Bio12: annual precipitation (mm/year); Bio15: precipitation seasonality (variant coefficient); Bio17: precipitation of driest quarter (mm/quarter); Bio18: precipitation of warmest quarter (mm/quarter); Bio19: mean monthly precipitation amount of the coldest quarter (mm/quarter). The initials for the type of behavior correspond to E = Extrapolation, C = Clamping, NE = No Extrapolation. Soil cover values 7, 9 and 18 correspond to shrub, herbaceous with sparce tree/shrub and urban respectively.

**Table A3**. Number of models that passed the different evaluation criteria applied in the calibration for the three reservoirs.

| Species | All candidate models | SS models | Models meeting OR | Models meeting AICc | SS models meeting OR | SS models meeting AICc | SS models meeting OR and AIICc |
| --- | --- | --- | --- | --- | --- | --- | --- |
| *O. chacoensis* | 1581 | 1581 | 676 | 1 | 676 | 1 | 1 |
| *O. f. occidentalis* | 1581 | 1581 | 987 | 3 | 987 | 3 | 1 |
| *C. fecundus* | 1581 | 1581 | 1053 | 1 | 1053 | 1 | 1 |

Note: SS = Statistically Significant, AICc = Akaike Informative Criterion corrected, OR = Omission Rate.

**Table A4**. Selected models according to the three defined criteria of selection: Partial ROC, Omission Rates and Akaike Informative Criteria corrected (AICc).

| Specie | Occurrence records | Train | Test | Selected Model | Partial ROC | Omission Rates (<5%) | AICc | ΔAICc |
| --- | --- | --- | --- | --- | --- | --- | --- | --- |
| *O. chacoensis* | 106 | 84 | 22 | M_2_F_lpt_Set_1 | 0 | 0.045 | 2416.15 | 0 |
| *O. f. occidentalis* | 99 | 79 | 20 | M_4_F_lqt_Set_3 | 0 | 0.05 | 2252.794 | 0 |
| *C. fecundus* | 174 | 139 | 35 | M_2_F_qpt_Set_3 | 0 | 0.029 | 3638.635 | 0 |

Note: The values of these three parameters are better when approaching to 0.


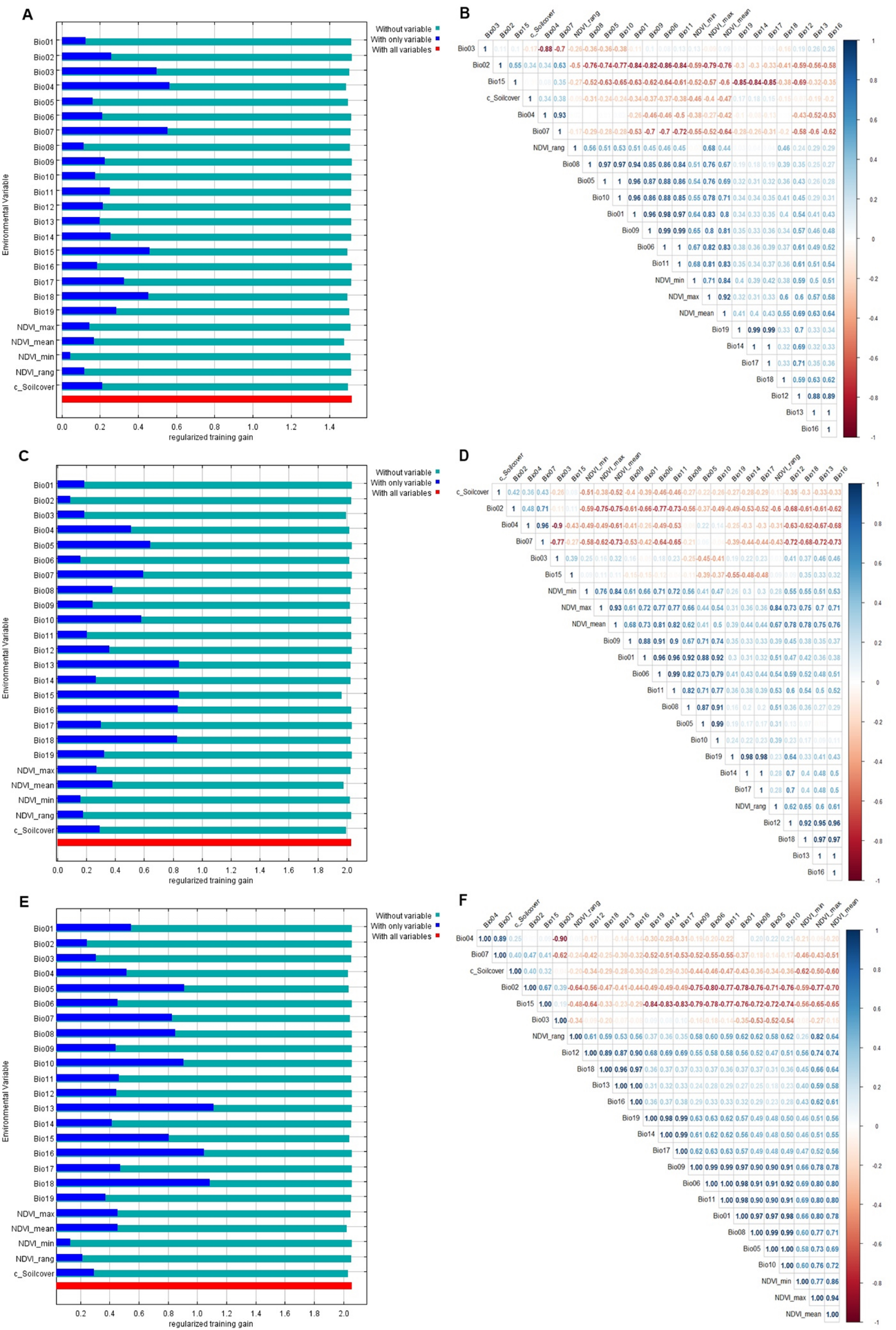


**Fig. A1**. Jackknife analysis and Pearson test in M area for the three reservoirs. *O. chacoensis* (A and B), O*. f. occidentalis* (C and D) and *C. fecundus* (E and F).

**
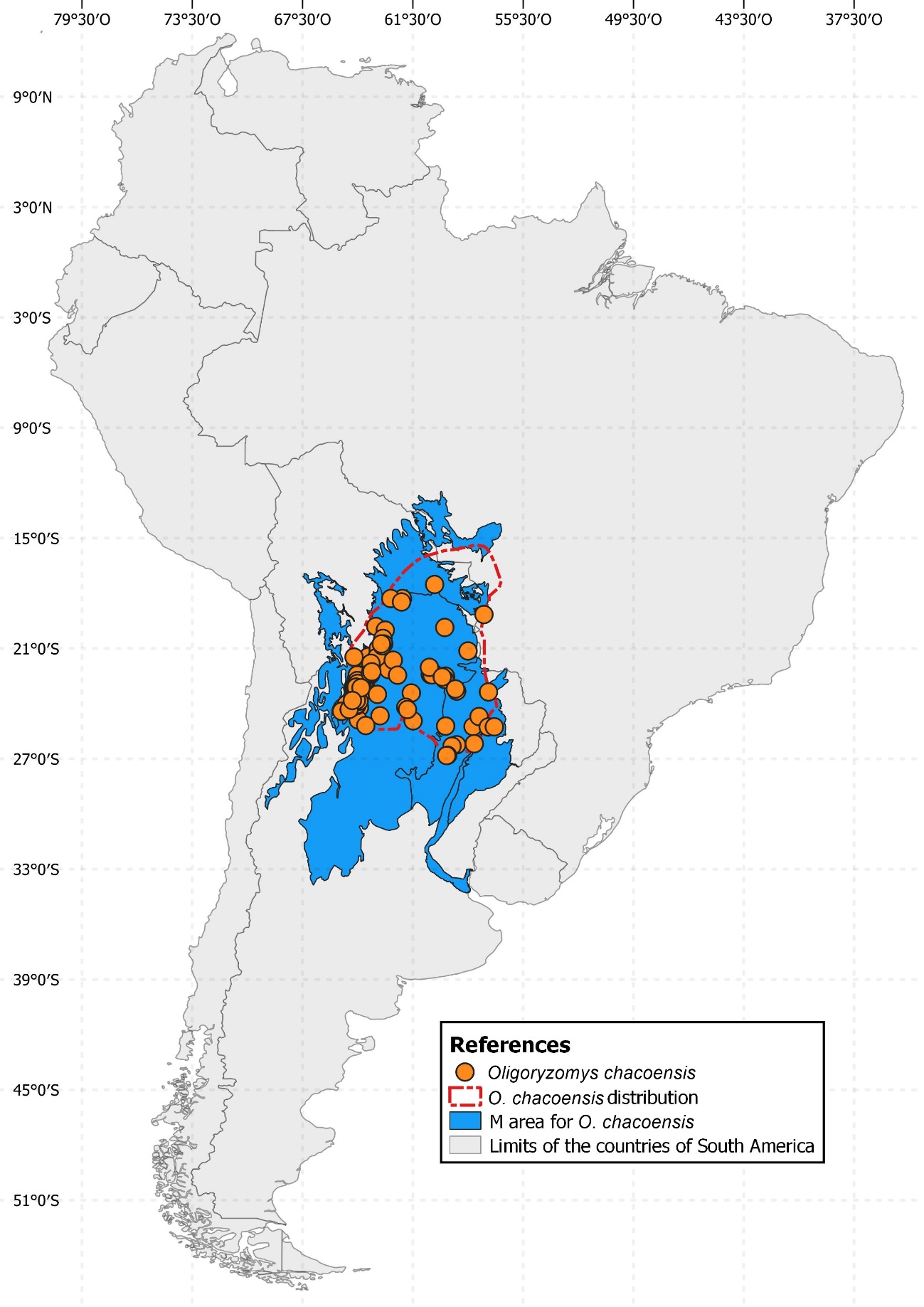
**

**Fig. A2**. Points of occurrence of *O. chacoensis* in South America. This figure was created in QGIS V.3.20.2, using free and freely available shapefiles.

**
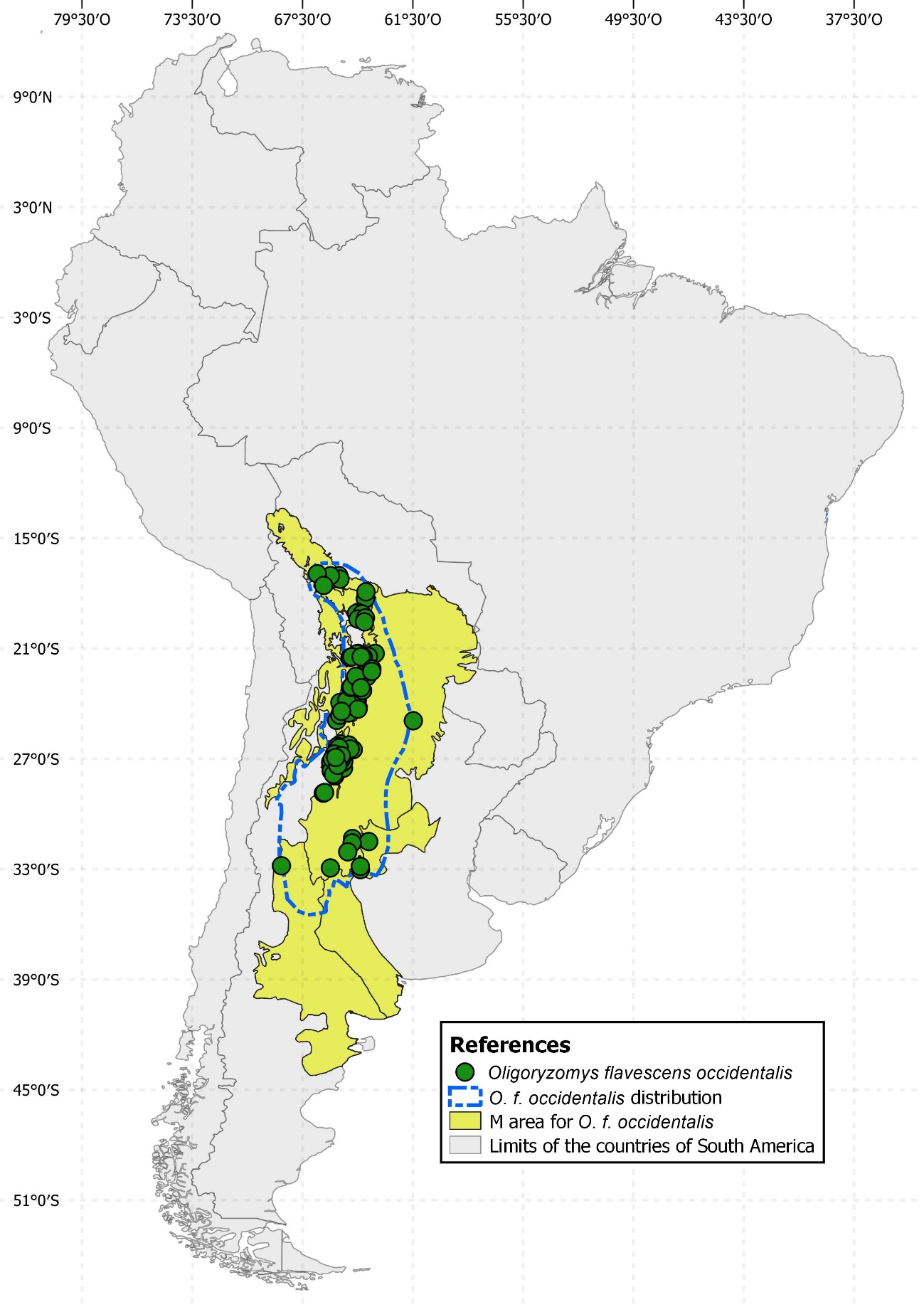
**

**Fig. A3**. Points of occurrence of *O. f. occidentalis* in South America. This figure was created in QGIS V.3.20.2, using free and freely available shapefiles.

**
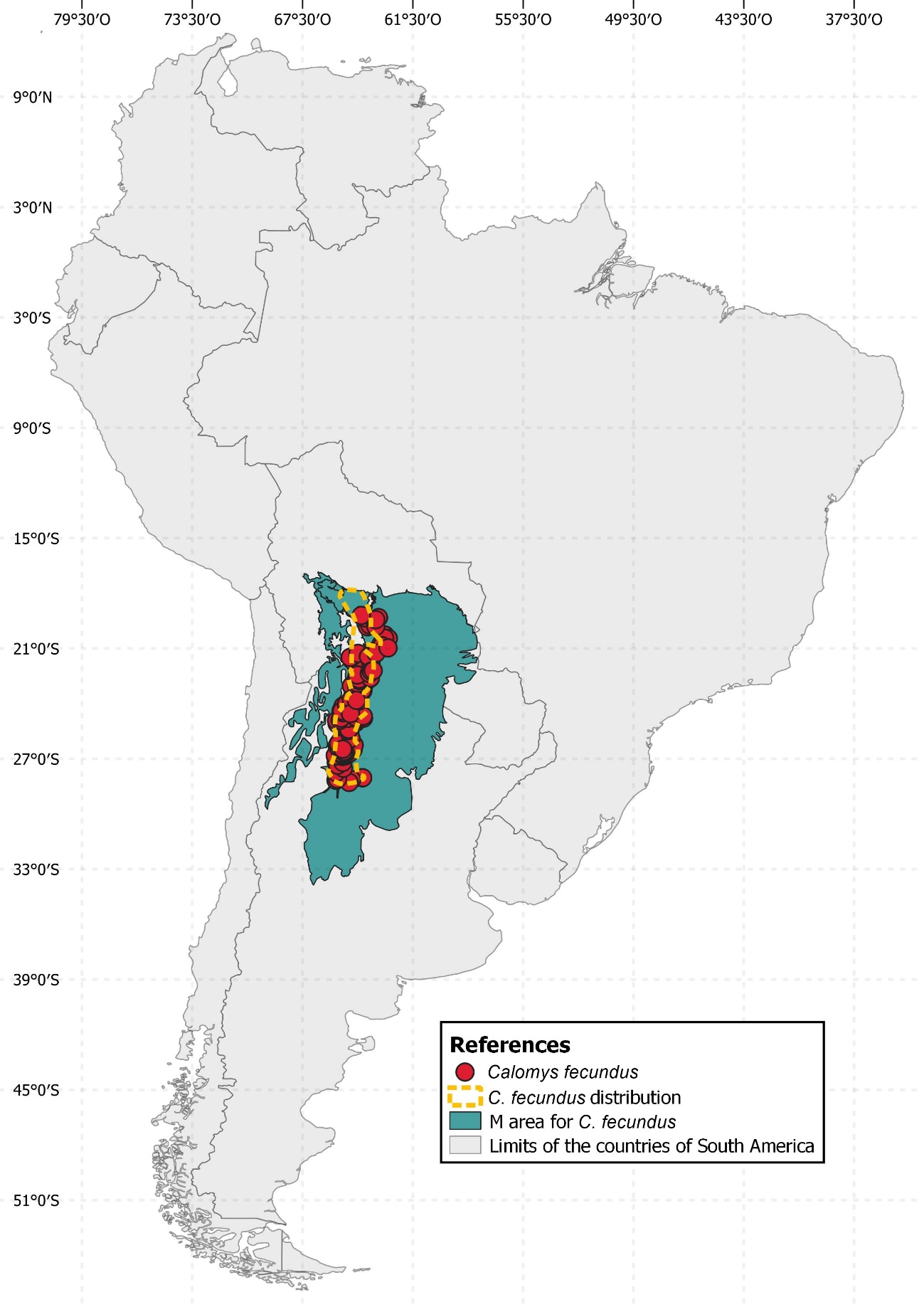
Fig. A4**. Points of occurrence of *C. fecundus* in South America. This figure was created in QGIS V.3.20.2, using free and freely available shapefiles.


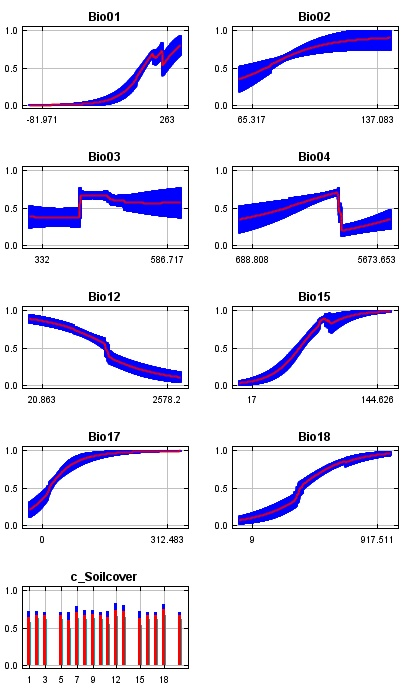


**Fig. A5**. Response curve for *O. chacoensis*.


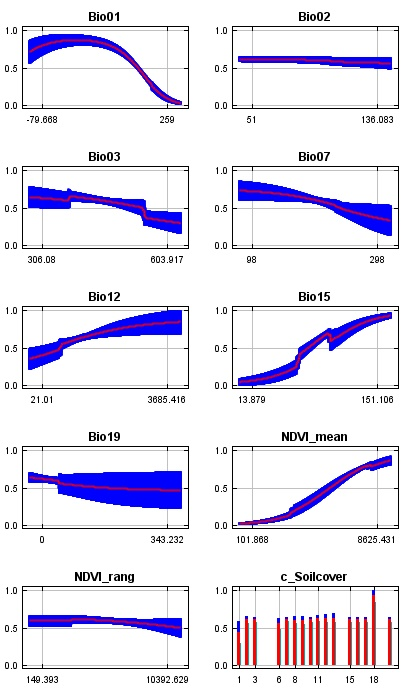


**Fig. A6**. Response curve for *O. f. occidentalis*.


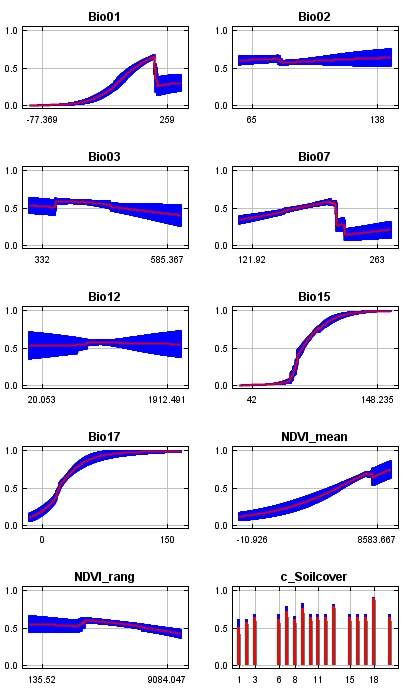


**Fig. A7**. Response curve for *C. fecundus*.


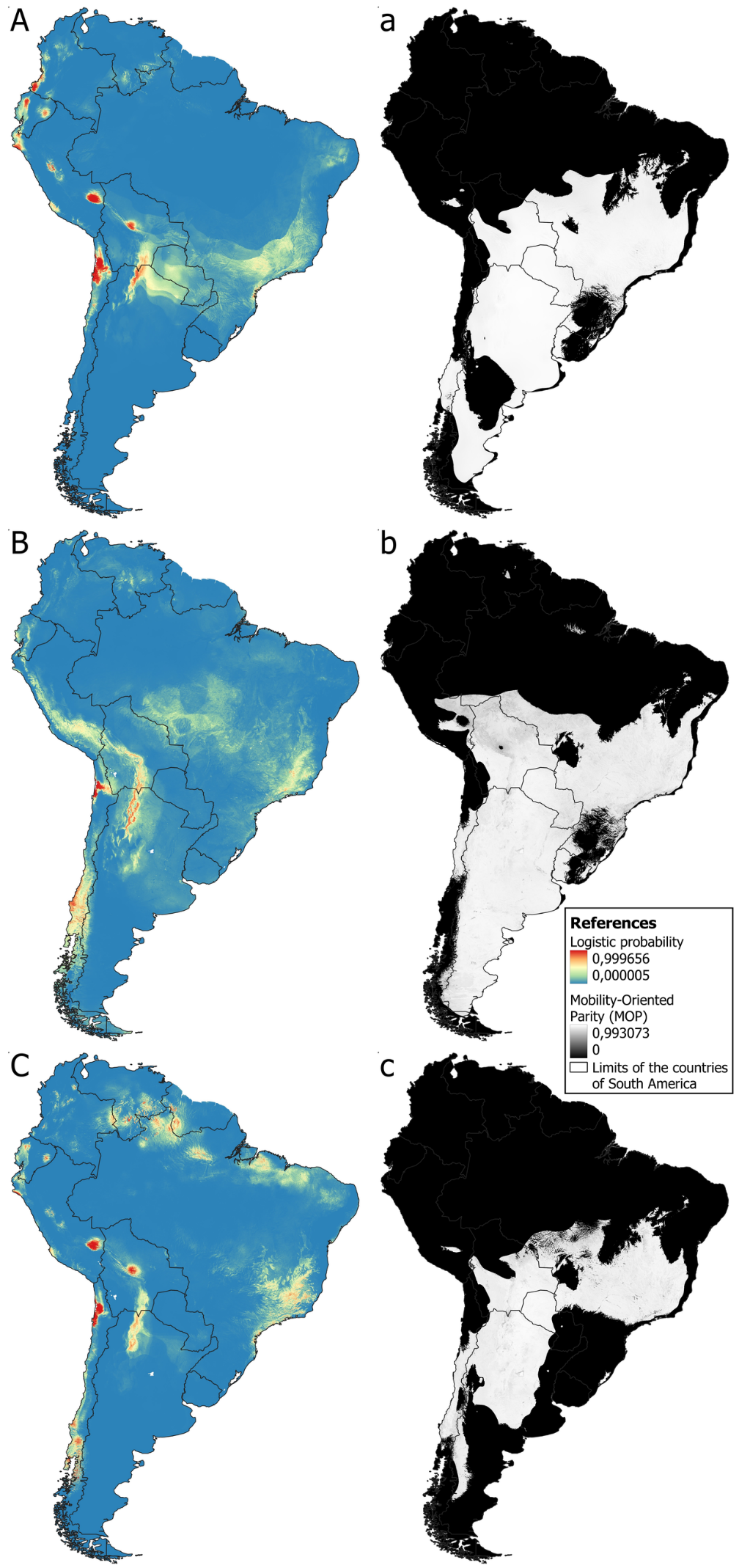


**Fig. A8**. Logistic outputs of best models extrapolated in South America, and MOP analysis for the three reservoirs. *O. chacoensis* (A, a), *O. f. occidentalis* (B, b) and *C. fecundus* (C, c). This figure was created in QGIS V.3.20.2, using free and freely available shapefiles.

**References**

- Porto Tapiquen, CE. Paises del Mundo. Orogénesis Soluciones Geográfica. Porlamar, Venezuela. Basado en capas de Enviromental Systems Research Institute (ESRI). Distribución Gratuita. 2015. <https://www.efrainmaps.es/descargas-gratuitas/mundo/>.
- Instituto Geográfico Nacional de la República Argentina. Departamentos de la República Argentina (shapefile). 2019. <https://www.ign.gob.ar/NuestrasActividades/InformacionGeoespacial/CapasSIG>.
- Chamberlaing S, Boettiger C. R Python, and Ruby clients for GBIF species occurrence data. PeerJ. 2017;1-32. https://doi.org/10.7287/peerj.preprints.3304v1.
- *Oligoryzomys chacoensis* (Myers & Carleton, 1981) in GBIF Secretariat. 2019. GBIF Backbone Taxonomy. GBIF.org (12 November 2019) GBIF Occurrence Download https://doi.org/10.15468/dl.tsqzyk. Accessed from R via rgbif (https://github.com/ropensci/rgbif) on 2019-11-12.
- *Oligoryzomys flavescens* (Waterhouse, 1837) in GBIF Secretariat. 2019. GBIF Backbone Taxonomy. GBIF.org (12 November 2019) GBIF Occurrence Download https://doi.org/10.15468/dl.kzz4sb. Accessed from R via rgbif (https://github.com/ropensci/rgbif) on 2019-11-12.
- *Calomys boliviae* (Thomas, 1901) in GBIF Secretariat. 2020. GBIF Backbone Taxonomy. GBIF.org (14 September 2020) GBIF Occurrence Download https://doi.org/10.15468/dl.whhs6z. Accessed from R via rgbif (https://github.com/ropensci/rgbif) on 2020-09-14.
- Zizka A, Silvestro D, Andermann T, Azevedo J, Ritter CD, Edler D, Farooq H, et al. CoordinateCleaner: Standardized cleaning of occurrence records from biological collection databases. Methods in Ecology and Evolution. 2018;10(5):744-751. https://doi.org/10.1111/2041-210X.13152.
- IUCN (International Union for Conservation of Nature). 2008. *Oligoryzomys chacoensis*. The IUCN Red List of Threatened Species. Version 2021-3. https://www.iucnredlist.org/species/15243/115126239. Accessed 8 June 2021.
- International Union for Conservation of Nature (IUCN). 2008. *Oligoryzomys flavescens*. The IUCN Red List of Threatened Species. Version 2021-3. https://www.iucnredlist.org/species/15247/115126612. Accessed 8 Jun 2021.
- International Union for Conservation of Nature (IUCN). 2008. *Calomys fecundus*. The IUCN Red List of Threatened Species. Version 2021-3. https://www.iucnredlist.org/species/136737/22335065. Accessed 8 Jun 2021.
- International Union for Conservation of Nature (IUCN). 2016. *Calomys boliviae*. The IUCN Red List of Threatened Species. Version 2021-3 <https://www.iucnredlist.org/species/3609/22334892>. Accessed 8 Jun 2021.
- Ortiz PE, Jayat JP. 2019. *Oligoryzomys chacoensis*. En: SAyDS–SAREM (eds.) Categorización 2019 de los mamíferos de Argentina según su riesgo de extinción. Lista Roja de los mamíferos de Argentina. https://cma.sarem.org.ar/es/especie-nativa/oligoryzomys-chacoensis. Accessed 8 Jun 2021.
- Trimarchi LI, González-Ittig RE, d´Hiriart S. 2019. *Oligoryzomys occidentalis*. En: SAyDS–SAREM (eds.) Categorización 2019 de los mamíferos de Argentina según su riesgo de extinción. Lista Roja de los mamíferos de Argentina. https://cma.sarem.org.ar/es/especie-nativa/oligoryzomys-occidentalis. Accessed 8 Jun 2021.
- González-Ittig RE, Pinotti JD, d´Hiriart S, Ortiz PE, Ferro LI. 2019. *Calomys boliviae*/*fecundus*. En: SAyDS–SAREM (eds.) Categorización 2019 de los mamíferos de Argentina según su riesgo de extinción. Lista Roja de los mamíferos de Argentina. https://cma.sarem.org.ar/es/especie-nativa/calomys-boliviae-fecundus. Accessed 8 Jun 2021.
- Busetto L, Ranghetti L. MODIStsp: An R package for automatic preprocessing of MODIS Land Products time series. Computers & Geosciences. 2016;97:1-16. https://doi.org/10.1016/j.cageo.2016.08.020.
- Hijmans RJ, van Etten J. raster: Geographic analysis and modeling with raster data. 2012. R package version 2.0-12. <http://CRAN.R-project.org/package=raster>.
- QGIS.org. QGIS Geographic Information System. QGIS Association. 2021. <http://www.qgis.org>.
- INDEC (Instituto Nacional de Estadística y Censos). 1991. Censo 1991. <https://www.indec.gob.ar/indec/web/Nivel4-Tema-2-41-136>. Accessed 1 May 2022.
- INDEC (Instituto Nacional de Estadística y Censos). 2001. Censo 2001. https://www.indec.gob.ar/indec/web/Nivel4-Tema-2-41-134. Accessed 11 May 2022.
- INDEC (Instituto Nacional de Estadística y Censos). 2010. Censo 2010. https://www.indec.gob.ar/indec/web/Nivel4-Tema-2-41-135. Accessed 11 May 2022.
